# Reduction of RF Heating Near Bilateral Deep Brain Stimulation Leads Using Two‐Channel RF Shimming at 3T

**DOI:** 10.1002/nbm.70129

**Published:** 2025-08-31

**Authors:** C. D. E. van Speybroeck, W. Roskamp, E. Arts, R. S. Vinke, M. van der Graaf, W. M. Brink

**Affiliations:** ^1^ Department of Medical Imaging Radboud University Medical Center Nijmegen the Netherlands; ^2^ Donders Institute for Brain, Cognition and Behaviour, Department of Neurosurgery Radboud University Medical Center Nijmegen the Netherlands; ^3^ Multi‐Modality Medical Imaging Group, TechMed Center University of Twente Enschede the Netherlands

**Keywords:** deep brain stimulation, MRI, radiofrequency heating, safety

## Abstract

The use of 3T MRI in patients with bilateral deep brain stimulation (DBS) leads is limited by safety concerns due to radiofrequency (RF) heating. A promising strategy to overcome this problem involves RF shimming using low‐specific absorption rate (SAR) calibration scans to estimate the RF‐induced currents based on image artifacts near the leads. Although clinically available two‐channel RF shimming can suppress RF heating in a single lead configuration, complete nulling is not possible when more than one lead is involved. This study aims to develop a method to minimize rather than null RF heating and optimize imaging performance during 3T MRI in a bilateral DBS lead configuration by using two‐channel RF shimming. An anthropomorphic phantom equipped with bilateral DBS leads and fiber‐optic temperature sensors was constructed. Optimal RF shim settings were determined in multiple phantom orientations using a low‐SAR calibration protocol. These settings were evaluated and compared with the quadrature mode by measuring local RF heating during a high‐SAR imaging sequence and inspecting residual image artifacts. Measured heating curves and imaging data confirmed that tailored RF shim settings minimized RF heating and image artifacts for both leads simultaneously in all orientations studied. Two‐channel RF shimming on a clinical 3T MRI scanner can thus be optimized in a bilateral DBS lead configuration to minimize RF heating and maximize imaging performance. This workflow could potentially enable a patient‐specific workflow for safe imaging in patients with bilateral DBS leads at 3T.

## Graphical abstract

This study addresses the RF heating risk of 3T MRI in patients with bilateral DBS leads. Using two‐channel RF shimming and low‐SAR calibration scans, we developed a method to minimize‐rather than eliminate—RF heating in both leads simultaneously. Optimized shim settings were shown to reduce RF heating and image artifacts in phantom experiments across multiple phantom orientations. This approach improved safety and imaging performance, suggesting a potential path toward safe imaging in patients with bilateral DBS leads at 3T.

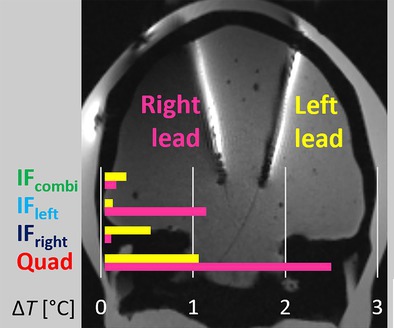

**Abbreviations**
CV: coefficient of variation
DBS: deep brain stimulation
FSE: fast spin echo
GRE: gradient‐recalled echo
IF: implant friendly
IPG: implantable pulse generator
RF: radiofrequency
SAR: specific absorption rate

## 1 Introduction

Deep brain stimulation (DBS) is a well‐established treatment for a variety of diseases, such as Parkinson's disease, tremor, dystonia, and even neuropsychiatric diseases [1, 2, 3]. In DBS treatment, one or multiple multielectrode leads are implanted in the brain and connected to an implantable pulse generator (IPG), which delivers electrical stimulation to specific deep brain structures to control symptoms. In Parkinson's disease, treatment typically involves bilateral implantation of DBS electrodes in both subthalamic nuclei [4]. These target anatomical structures can be described as ellipsoids, each with an approximate size of 5 × 5 × 10 mm [5]. Because of their small size, precise electrode placement within the nuclei is both challenging and crucial. Accurate positioning is essential, as it is associated with improved long‐term outcomes, whereas suboptimal placement can diminish treatment efficacy and increase adverse effects [6, 7, 8].

Planning of the DBS lead trajectories is typically based on a preoperative 3T MRI, which offers superior soft‐tissue contrast and spatial resolution for identification of the target neuroanatomical structures and insertion pathway. Verification of the lead placement typically involves coregistration of a postoperative CT with the preoperative MRI, which can introduce registration errors in the order of 1.5 mm [9, 10, 11]. Intraoperative or postoperative MR imaging, preferably at 3T, could enable direct verification of the lead placement relative to the visualized target, as well as probe functional parameters to assess treatment effectiveness [12, 13].

Radiofrequency (RF) heating of tissue, however, poses a significant safety concern when performing MRI on patients with DBS leads [14, 15]. As the RF transmit field can induce RF currents along the DBS lead, this can result in elevated energy dissipation and associated heating of brain tissues around the tip of the lead [16]. In addition, the magnetic field distortion generated by the RF currents results in image artifacts appearing around the lead [17]. Currently, most DBS systems are classified as MR‐unsafe at 3T. Many of these systems have received MR‐conditional labeling for scanning at 1.5T, under strict constraints on RF exposure in terms of *B*_1_^+^_,rms_ and/or specific absorption rate (SAR). Additionally, permitted scanning configurations are limited to either the complete system or the leads alone, explicitly excluding scenarios such as scanning DBS leads connected solely to extension cables.

Several studies have shown an increased variability in RF heating around DBS implants at higher *B*_0_ field strengths, making it challenging to establish generic safety constraints without being overly restrictive [18, 19, 20, 21, 22, 23, 24, 25, 26, 27, 28, 29, 30, 31, 32]. In general, RF nonuniformity increases because of the shortening of the RF wavelength as the *B*_0_ field strength increases, and is accompanied by increases in RF power absorption (considering a fixed *B*_1_^+^ level) [19, 20, 21]. Some studies have reported substantial RF heating at 3T under specific conditions, which would be anticipated based on the increased RF frequency [22, 23, 24, 25]. Conversely, other studies demonstrated minimal RF heating in specific configurations at 3T [26, 27, 28, 29, 30, 31, 32], with some even suggesting the possibility of off‐label scanning [32].

A promising strategy in this context involves using low‐SAR gradient‐recalled echo (GRE) imaging to characterize the RF‐induced current in an elongated conductive structure, such as a guidewire or a DBS lead [33, 34, 35, 36, 37, 38]. These methods leverage the characteristic geometry of the resulting image artifact to estimate the magnitude and phase of the associated current, which is in turn linked to the RF heating around the tip. The aforementioned information can be used to determine the optimal RF shimming regarding minimizing the RF‐induced current. Specifically, two‐channel RF shimming has been shown to allow mitigating the RF‐induced current on a single DBS lead completely by exploiting the two degrees of freedom (e.g., relative amplitude and relative phase) provided by a two‐channel RF transmit body coil [37, 38]. However, completely nulling the RF‐induced currents on a bilateral lead configuration is known to require a higher number of RF transmit channels [39, 40, 41]. Such advanced RF architectures are typically not available on clinical 3T MRI systems, limiting the utility of this approach in clinical settings.

The goal of this study is to extend the aforementioned strategy of using the characteristic artifact to minimize, rather than null, the induced current in a bilateral DBS configuration using two‐channel RF shimming at 3T. By using a low‐SAR calibration procedure, we determine the optimal RF settings that minimize the RF‐induced currents in both leads simultaneously while maximizing the available *B*_1_^+^ field strength to preserve image quality. The procedure and its robustness are validated using temperature recordings in an anthropomorphic phantom equipped with bilateral DBS leads and comparing the optimal RF settings to a standard quadrature excitation.

## 2 Methods

All experiments were conducted using a 3T MRI system (MAGNETOM Skyra, Siemens Healthineers, Erlangen, Germany) equipped with a two‐channel integrated RF transmit body coil and a 20‐channel head coil (Head/Neck 20, Siemens Healthineers, Erlangen, Germany) for signal reception.

**FIGURE 1:**
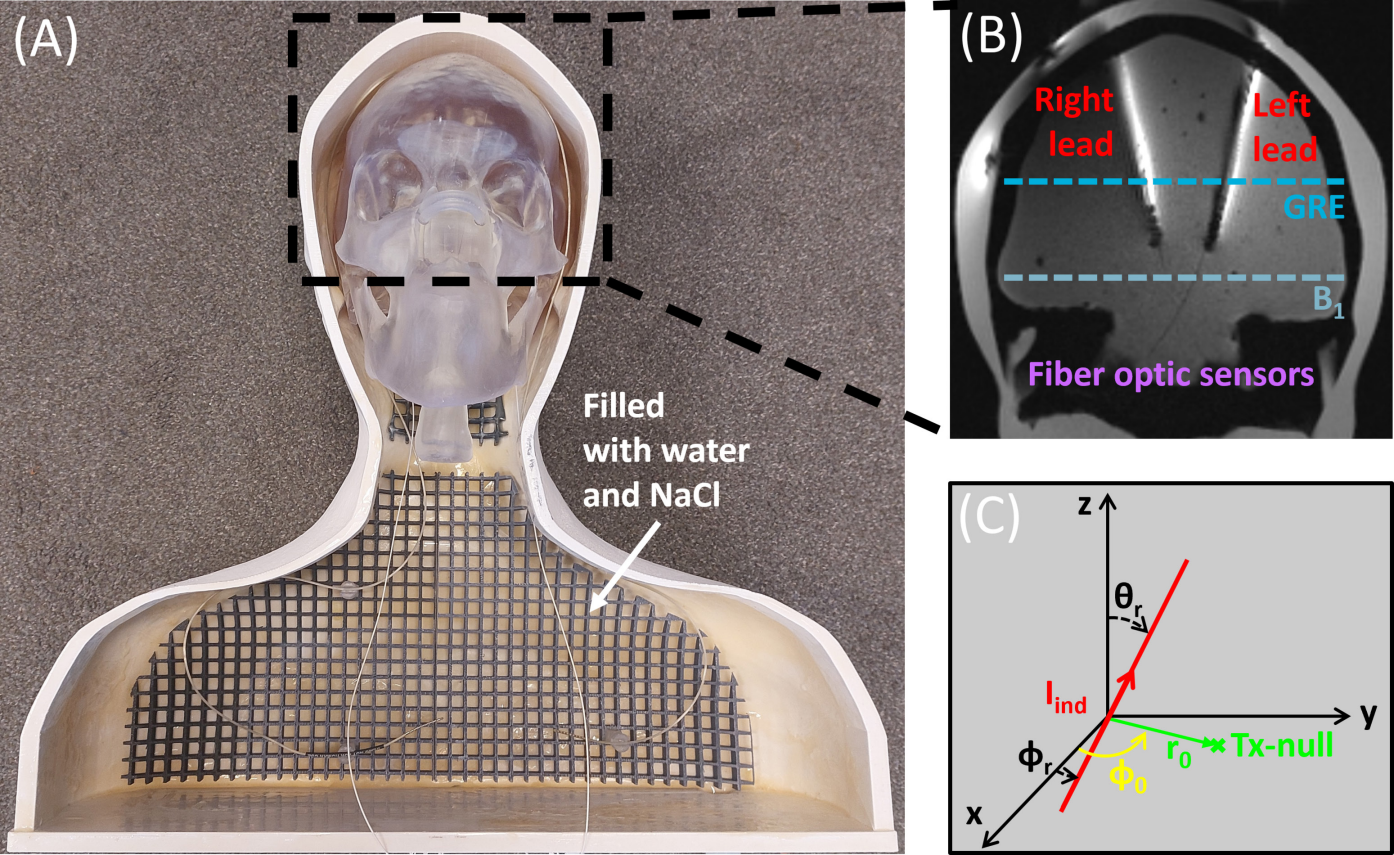
Experimental setup with the anthropomorphic phantom. (A) A photograph of the anthropomorphic phantom, indicating the human‐shaped torso and skull, of which the skull compartment was filled with an aqueous gel ($\varepsilon_{r}$ = 53.6 and σ = 0.35 S/m) and the torso compartment was filled with a saline solution ($\varepsilon_{r}$ = 78 and σ = 0.50 S/m). (B) A magnetic resonance coronal image showing the position of the deep brain stimulation leads, positioned on the patient‐right side and patient‐left side, respectively, and the fiber optic temperature probes. In the image, the position of the low‐SAR gradient‐recalled echo (GRE) calibration scan and the *B*_1_ map is also indicated by a dashed line. (C) The lead orientation and configuration at polar and azimuthal angles $\theta_{r}$ and $\phi_{r}$. The induced current and the position of the Tx‐null artifact with respect to the lead are also indicated by the radius *r*_0_ and phase *φ*_0_.

Experiments were performed in an anthropomorphic phantom, which consisted of a skull and torso compartment [24], shown in Figure 1A. The skull was constructed using a stereolithography 3D printer (Clear Photopolymer Resin V4, Formlabs, Berlin, Germany), and the torso was constructed using regular fused deposition modeling (ABS MX4, Urbicum, Zielonki, Poland). The interior of the torso compartment was coated with epoxy to ensure that it was leak‐proof. Two DBS leads (Vercise Cartesia Directional, DB‐2202, Boston Scientific, Marlborough, MA) were positioned in the skull compartment to mimic bilateral placement in the subthalamic nuclei. The leads were inserted into the skull phantom at anatomically appropriate entry points and positioned to a depth of 9.5 cm relative to the outer surface of the skull. The trajectory of each lead was angulated at 12° in the right–left direction and 5° in the anterior–posterior direction, simulating a clinically relevant implantation path. Two fiber optic temperature probes (OTP‐M series, Opsens, Quebec City, QC) were bound to each lead tip, corresponding to the location of maximum heating, as illustrated in Figure 1B [42]. The skull compartment was then filled with an aqueous gel to approximate the average dielectric properties of white matter, consisting of 70‐g polyvinylpyrrolidone gel (PVP10, Sigma Aldrich, the Netherlands), 1‐g sodium chloride (NaCl), and 2.6‐g agarose (A9539, Sigma Aldrich, the Netherlands) per 100 g of demineralized water ($\varepsilon_{r}$ = 53.6 and σ = 0.35 S/m) [43, 44]. The torso compartment was filled with a saline solution of 2.25‐g/L NaCl in demineralized water ($\varepsilon_{r}$ = 78 and σ = 0.50 S/m) [24].

Following the phantom preparation, the exact location and orientation of the leads were determined using a low‐SAR multislice coronal GRE sequence (TR/TE = 262/5.19 ms, flip angle = 10°, field‐of‐view = 256 × 256 mm, in‐plane spatial resolution = 1 × 1 mm, slice thickness = 2 mm, number of slices = 12, acquisition time = 69 s, *B*_1_^+^_,rms_ = 0.09 μT), as shown in Figure 1B. The slice containing the leads was then used to estimate the angle of the lead with respect to the *B*_0_ field, $\theta_{r}$, as illustrated in Figure 1C. As a multislice sequence was used, $\phi_{r}$ could also be estimated from the images.

RF shim settings were then designed based on a series of low‐SAR GRE acquisitions, extending the method of Eryaman et al. [38]. This approach leverages the characteristic geometrical properties of the image artifact that is produced around a conductive wire when the secondary *B*_1_^+^ field, produced by an RF current induced on the lead, cancels the primary or incident *B*_1_^+^ field, which is generated by the RF transmit coil. The location of this point of signal cancellation, hereafter referred to as “Tx‐null,” with respect to the location of the lead, is described by the radius $r_{0}$ and phase $\phi_{0}$ as defined in Figure 1C. Together with the polar and azimuthal angles $\theta_{r}$ and $\phi_{r}$ of the lead, this location can be directly related to the magnitude and phase of the RF current that was induced on the associated lead, as described in [42, 45], using the following equation:

(1) $$I=\frac{4\pi r_{0}}{\mu_{0}}\sqrt{\cos^{2}\theta_{r}\cos^{2}\left(\phi_{0}-\phi_{r}\right)+\sin^{2}\left(\phi_{0}-\phi_{r}\right)}\left|B_{1,\mathrm{inc}}^{+}\right|$$

**FIGURE 2:**
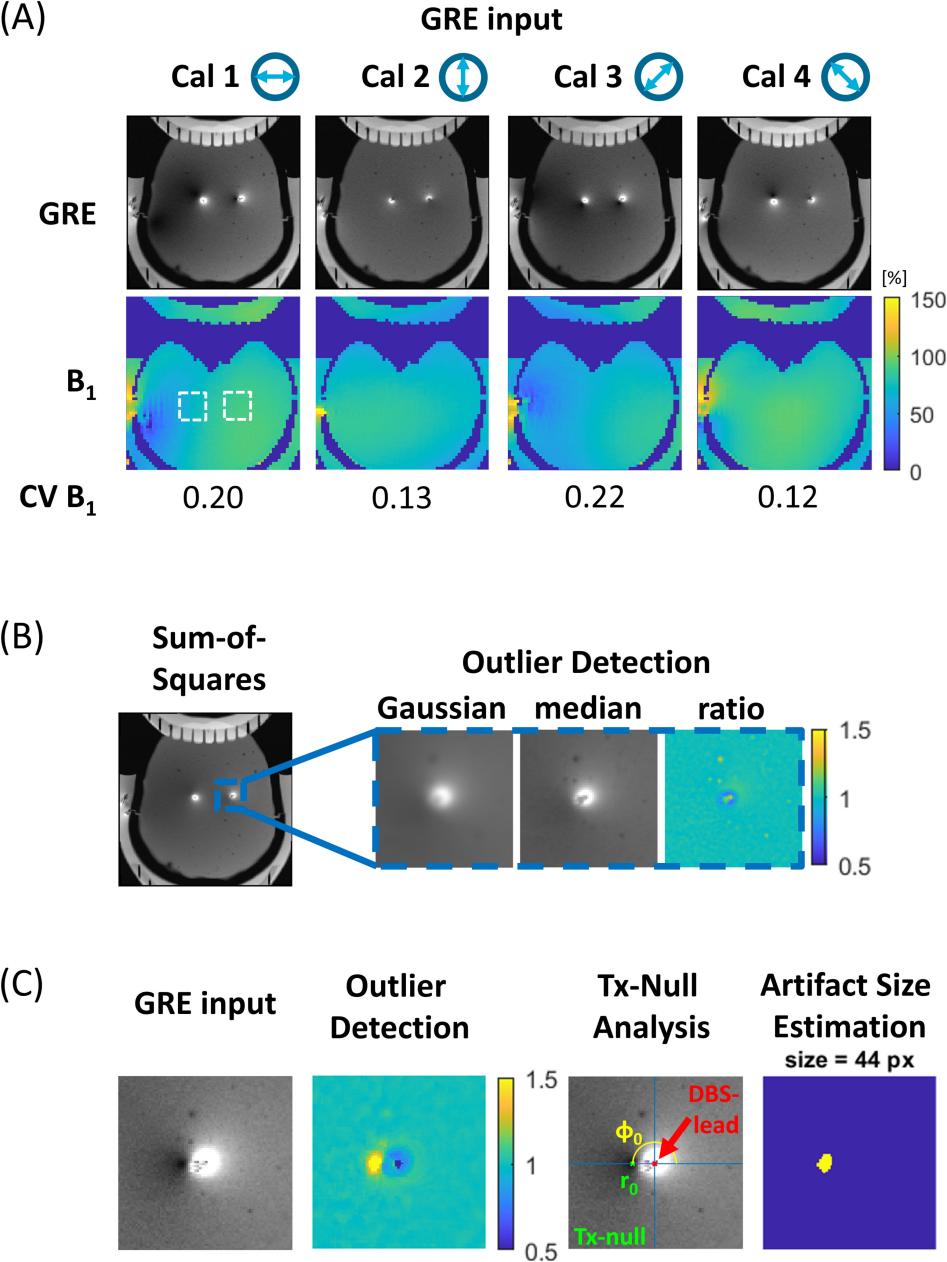
(A) Four low‐SAR gradient‐recalled echo (GRE) calibration scans and their *B*_1_ maps and corresponding coefficient of variation (CV). Their respective linear incident *B*_1_^+^ field polarizations are indicated by the arrow. The patient right lead is indicated on the left and vice versa. The left *B*_1_ map has the incident *B*_1_^+^ fields of both leads indicated by a white square. (B) Methodology for analyzing the calibration scans. Left: Sum‐of‐squares combination of the four GRE calibration scans used for outlier detection. Right: Outlier detection method, where Gaussian filtered and median filtered images are pixelwise divided to establish the lead position. (C) Method for determining the relative position of the Tx‐null artifact with respect to the lead position and the size of the artifact, based on the method depicted in (B).

Here, $\left|B_{1,\mathrm{inc}}^{+}\right|$ denotes the magnitude of the incident *B*_1_^+^ field, which can be measured below the lead tip, directly underneath the Tx‐null location, as indicated in Figures 1B and 2A. It is important to note that in this study, the Tx‐null was defined in the axial plane orthogonal to the *B*_0_ field direction, as opposed to defining it in a plane orthogonal to the lead [45]. Strictly speaking, this approach preserves the *relative* phase and amplitude of both currents (i.e., which is relevant to the design of the RF shim), only when the bilateral lead configuration is positioned symmetrically with respect to the *B*_0_ field direction. We will therefore assess the method in rotated configurations as well to test this approximation.

To characterize the RF‐induced current on the DBS leads for each of the two RF transmit channels, the locations of the Tx‐null artifacts were determined in four different linear polarizations of the incident *B*_1_^+^ field. Since the DBS lead can potentially reside in the *E*‐field null‐plane of a linearly polarized transmit field [46, 47], employing four different RF polarizations ensured that a nonzero (i.e., measurable) current was induced in at least three out of four datasets. The RF shim settings corresponding to each of the four RF polarizations, as depicted in Figure 2A, are detailed in Table 1.

**TABLE 1:** RF shim settings with the relative amplitudes and phases of different linear incident *B*_1_^+^ field polarizations for the calibration scans.

	Channel 1	Channel 2
Calibration 1	1.00 ∠0°	0.00 ∠0°
Calibration 2	0.00 ∠0°	1.00 ∠0°
Calibration 3	0.71 ∠0°	0.71 ∠0°
Calibration 4	0.71 ∠0°	0.71 ∠180°
Quadrature	0.71 ∠0°	0.71 ∠90°

For each of the incident RF field polarizations, a low‐SAR single‐slice axial GRE sequence was acquired (TR/TE = 200/3.26 ms, flip angle = 30°, field‐of‐view = 160 × 160 mm, in‐plane spatial resolution = 0.5 × 0.5 mm, slice thickness = 6 mm, acquisition time = 36 s, *B*_1_^+^_,rms_ = 0.54 μT) positioned 30 mm above the lead tip, in conjunction with a single‐slice axial *B*_1_^+^ map based on a low‐SAR presaturated TurboFLASH sequence (TR/TE = 5000/2.37 ms, flip angle = 8°, slice thickness = 10 mm, acquisition time = 10 s) positioned 10 mm below the lead tip [48]. Both locations are shown in Figure 1B. Their images are illustrated in Figure 2A, which also reports the inhomogeneities present in the incident *B*_1_^+^ fields. These inhomogeneities are quantified using the coefficient of variation (CV), calculated over the entire transverse cross‐section of the phantom. The measurements were taken at these specific locations to avoid interference from susceptibility‐related artifacts around the lead electrodes and to estimate the incident *B*_1_^+^ field magnitude in a region free from RF field perturbations caused by the DBS lead [38].

**FIGURE 3:**
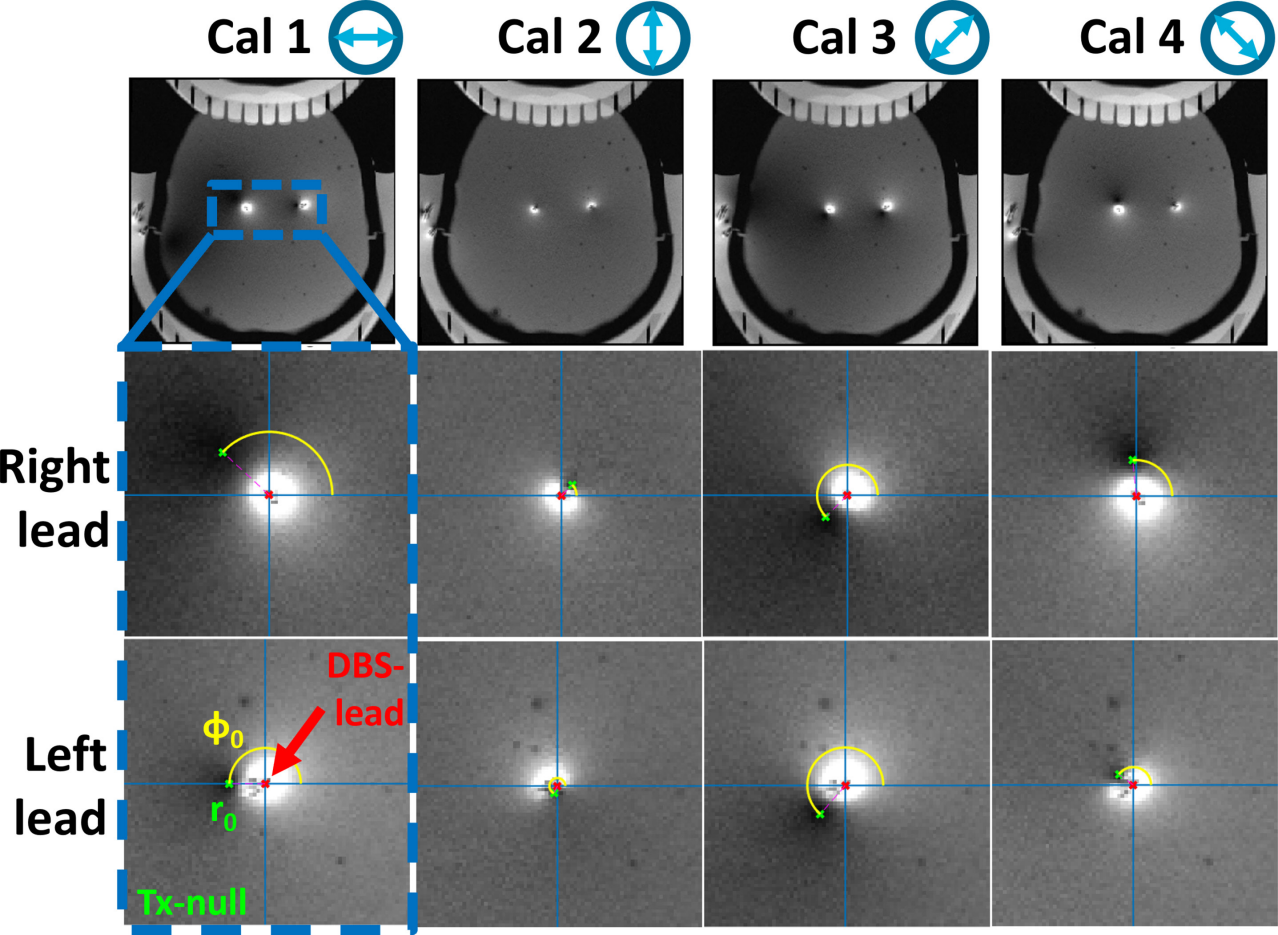
Location of the Tx‐null artifact (red dot) with respect to each lead, indicated by the radius *r*_0_ (green dot) and phase *φ*_0_ (yellow line), as determined from each of the gradient‐recalled echo calibration scans. Their respective linear incident *B*_1_^+^ field polarizations are indicated by the arrow. The patient right lead is indicated on the left and vice versa.

Figure 2B illustrates the semiautomatic method for analyzing the GRE images. The images were first combined in a sum‐of‐squares manner to mitigate the Tx‐null artifact and enable identification of the DBS lead positions. These were determined through a semiautomatic procedure for image outlier detection, where the user could provide approximate locations of the leads on the sum‐of‐squares image, shown left in Figure 2B [49, 50]. The procedure was based on a pixelwise division of a Gaussian filtered and a median filtered image, both using a kernel size of 5 pixels, after which a clear local maximum can be detected at the location of the DBS lead. Once both lead positions were established, the same procedure was used to determine the relative positions of the Tx‐null artifacts with respect to the associated leads. After excluding a circular region with a 1‐mm radius around the DBS lead locations, the processing steps were applied to all individual magnitude GRE images to determine the radii *r*_0_ and phases *φ*_0_. An additional step was taken to determine the Tx‐null artifact size by counting all pixels exceeding a ratio of 1.3 and taking the voxel size into account. An example of this procedure is illustrated in Figure 2C. The relative locations of the Tx‐null artifacts, as shown in Figure 3, were then used to determine the induced currents on the leads for each of the two RF transmit channels by inverting the associated interferometric equation, taking into account the polar and azimuthal angles $\theta_{r}$ and $\phi_{r}$ of the lead [45, 51]. Including all four incident field polarizations in this process enhanced robustness, ensuring reliability even when one of the induced currents was very low (e.g., showing negligible Tx‐null artifact).

After the RF interaction with the DBS and underlying *B*_1_^+^ sensitivity of the transmit coil was characterized, an RF shim was optimized to maximize the average *B*_1_^*+*^ field strength to improve image quality. At the same time, the maximum of two induced current magnitudes of either lead was minimized to minimize RF heating. The optimum RF shim was therefore determined by maximizing the *B*_1_^+^ efficiency as follows:

(2) $$\max\frac{\bar{B_{1}^{+}}}{\max\left(\left|I_{1}\right|,\left|I_{2}\right|\right)}$$

where $\bar{B_{1}^{+}}$ denotes the average *B*_1_^*+*^ magnitude measured across the transverse cross‐section and $\left|I_{1}\right|,\left|I_{2}\right|$ denotes the magnitude values of the induced currents on each lead, respectively [50]. This procedure minimized the dominant induced current, that is, the limiting parameter in terms of RF safety, while maximizing the available *B*_1_^+^.

The optimized RF shim that maximizes Equation (2) will be called the “combined implant friendly (IF) mode” abbreviated as IF_combi_, hereafter. RF shims that null the current on either lead were determined as well, following the analytic expressions of Eryaman et al., for comparison [38]. These modes will be called the implant‐friendly mode of the right lead and left lead, abbreviated as IF_right_ and IF_left_, respectively.

To verify the performance of the obtained RF shims, low‐SAR GRE images and *B*_1_^*+*^ maps were acquired with identical sequence parameters as used during calibration. The outlier detection procedure was used to characterize the residual Tx‐null artifact, including its size, and the related residual RF‐induced current on the lead. Additional data were acquired in quadrature mode to serve as a reference. Throughout the imaging experiments, temperature increases at both lead tips were monitored.

Using the previously determined shim settings for the IF_combi_, IF_right_, IF_left_, and quadrature modes, RF heating measurements were carried out using the fiber optic sensors during a high‐SAR fast spin echo (FSE) sequence (TR/TE = 3000/110 ms, refocusing flip angle = 150°, field‐of‐view = 250 × 219 mm, in‐plane spatial resolution = 0.8 × 0.8 mm, slices = 22, slice thickness = 2 mm, acquisition time = 314 s). The average RF power and *B*_1_^+^_,rms_ were recorded from the system's SAR monitor in all of the heating experiments. A cooling period of 10 min was accommodated between experiments to allow the phantom to return to a baseline temperature.

To test the orientation dependence of the method, the experiment was repeated with the phantom placed in two different orientations, at an angle of −3° and +10° with respect to the *B*_0_ field. This was done to mimic scenarios in which the patient's head is not perfectly aligned with the bore and to evaluate the robustness of the method when the leads are oriented at varying angles relative to the *B*_0_ field. The optimized RF shim settings for both leads combined as well as for each individual lead were determined in each orientation, and their performance was verified using the GRE calibration scans and compared with quadrature mode. In both orientations, the temperature measurements were repeated as well.

To assess the resulting image quality in a human brain, a healthy volunteer without DBS leads was scanned using the same MRI protocol. The volunteer provided informed consent prior to participation.

## 3 Results

**FIGURE 4:**
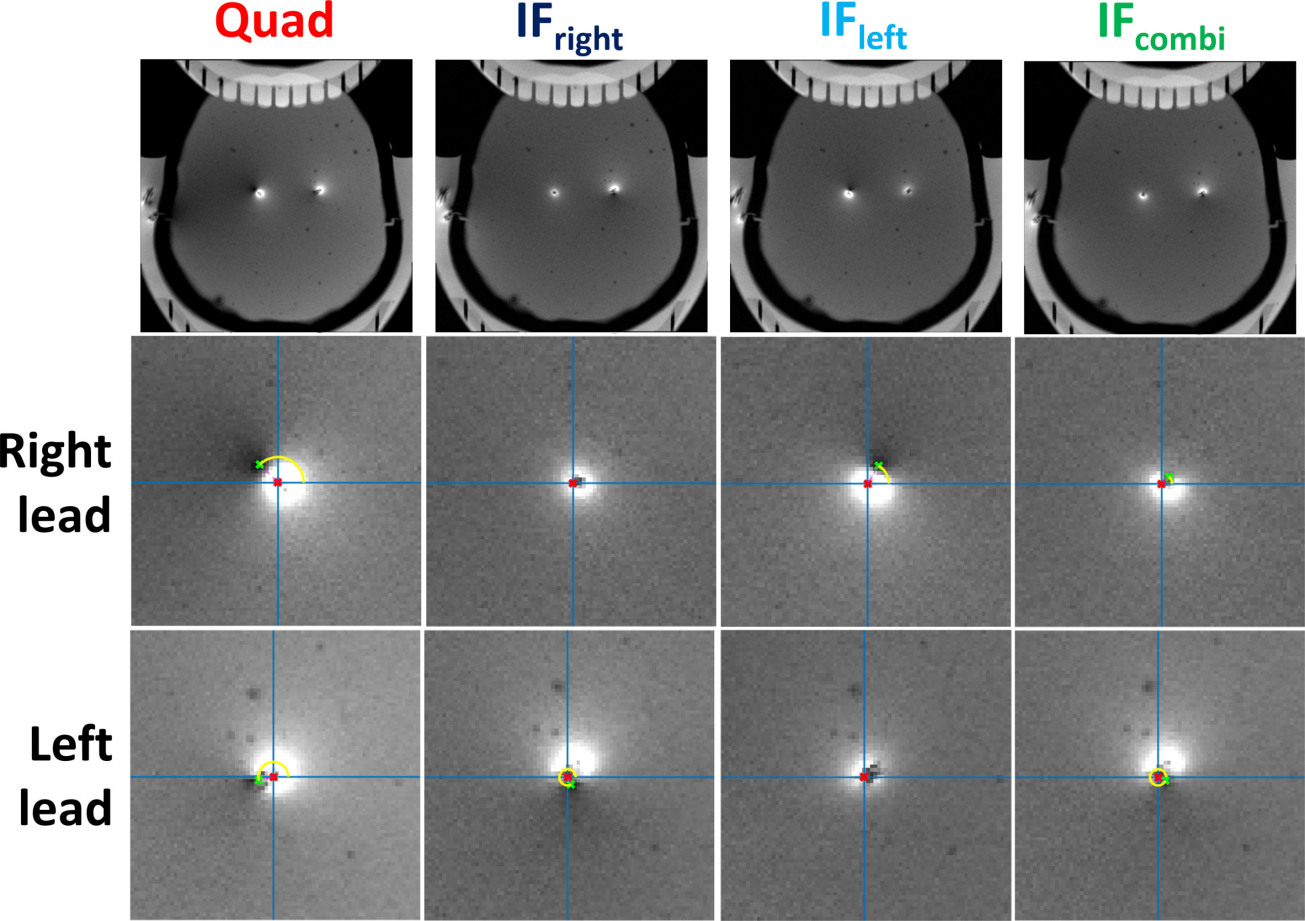
Location of the Tx‐null artifact (red dot) with respect to each lead, indicated by the radius *r*_0_ (green dot) and phase *φ*_0_ (yellow line), as observed in gradient‐recalled echo scans. Results are shown for quadrature mode (Quad), implant‐friendly modes of the right (IF_right_) and left lead (IF_left_), and the combined implant‐friendly mode (IF_combi_) from the gradient‐recalled echo scans. The patient right lead is indicated on the left and vice versa.

Figure 4 shows the low‐SAR GRE images of the obtained RF shims, for the quadrature, IF_right_, IF_left_, and IF_combi_ modes. In this figure, the residual Tx‐null artifact, if present, in the different modes is indicated for both leads. The specific RF shim settings of each transmit channel, used to scan the different modes and orientations shown in Figure 4, are detailed in Table 2.

**TABLE 2:** RF shim settings for each transmit channel in quadrature mode (Quad), the implant‐friendly modes of the right (IF_right_) and left lead (IF_left_), and the combined implant‐friendly mode (IF_combi_) across different orientations (O1–O3). For each mode and orientation, the RF power and *B*_1_^+^_,rms_ are provided. Additionally, the total temperature increase measured at each lead tip during high‐SAR fast spin echo scans is reported.

		Shim setting		RF power [W] / *B*_1_^+^_,rms_ [μT]	Temperature increase [°C]	
		Channel 1	Channel 2		Right lead	Left lead
O1	Quad	0.71 ∠0°	0.71 ∠90°	39.15/1.95	2.44 ± 0.01	1.00 ± 0.01
	IF_right_	0.23 ∠0°	0.97 ∠28°	34.17/1.65	−0.05 ± 0.01	0.44 ± 0.01
	IF_left_	0.20 ∠0°	0.98 ∠179°	33.71/1.63	1.09 ± 0.01	0.07 ± 0.01
	IF_combi_	0.13 ∠0°	0.99 ∠54°	33.57/1.61	0.11 ± 0.01	0.25 ± 0.01
O2	Quad	0.71 ∠0°	0.71 ∠90°	42.71/1.94	1.34 ± 0.01	3.24 ± 0.01
	IF_right_	0.64 ∠0°	0.77 ∠134°	40.85/1.84	0.63 ± 0.01	1.45 ± 0.01
	IF_left_	0.46 ∠0°	0.89 ∠‐145°	39.05/1.72	1.55 ± 0.01	−0.03 ± 0.01
	IF_combi_	0.53 ∠0°	0.85 ∠167°	39.33/1.74	0.68 ± 0.01	0.29 ± 0.01
O3	Quad	0.71 ∠0°	0.71 ∠90°	41.13/1.96	0.90 ± 0.01	6.96 ± 0.01
	IF_right_	0.48 ∠0°	0.88 ∠131°	37.17/1.75	0.16 ± 0.01	2.67 ± 0.01
	IF_left_	0.37 ∠0°	0.93 ∠‐123°	36.63/1.71	1.26 ± 0.01	0.01 ± 0.01
	IF_combi_	0.31 ∠0°	0.95 ∠‐159°	35.75/1.66	0.61 ± 0.01	0.31 ± 0.01

**FIGURE 5:**
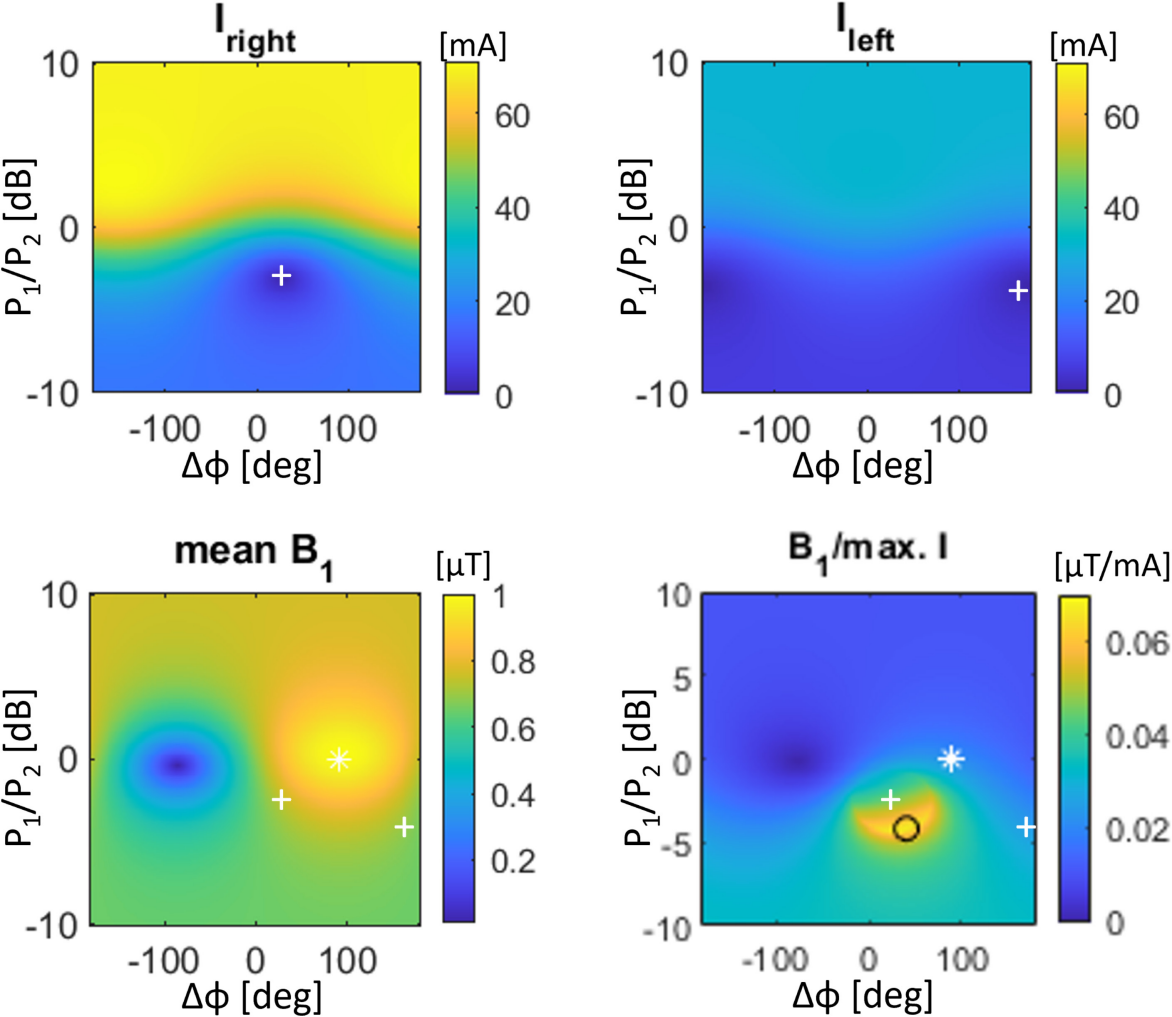
Variation of the induced current on the right lead, *I*_right_ (top left) and left lead, *I*_left_ (top right) as a function of RF shim parameters (i.e., relative power ratio *P*_1_/*P*_2_ and phase difference *Δφ*), in which the RF shim that null the induced current in either lead is indicated by a white plus (+). The mean *B*_1_^+^ (bottom left) relative to the largest of the two induced currents defines the *B*_1_^+^ efficiency (bottom right), both as a function of RF shim parameters. The bottom left and right figures indicate the earlier mentioned white plusses (+) and the conventional quadrature mode by the white star (*). The bottom right figure also indicates the RF shim that maximizes *B*_1_^+^ efficiency by the black circle (o).

Figure 5 shows an example of the variation of RF‐induced current on the right and left leads as well as *B*_1_^+^ magnitude, as a function of RF shim parameters (i.e., relative power ratio and RF phase difference). The *B*_1_^+^ efficiency, as a function of RF shim parameters, is obtained from the mean *B*_1_^+^ magnitude relative to the larger of the two induced currents. As a result, the RF shim settings for a unique optimum of the *B*_1_^+^ efficiency (IF_combi_) are obtained, which combines the minimization of the RF‐induced currents with consequent maximization of the *B*_1_^+^ magnitude to optimize image quality. In this case, the data confirm that the RF shim settings required to null the RF‐induced currents for either lead, corresponding to IF_right_ and IF_left_, respectively, do not coincide with the IF_combi_ mode. This confirms that both leads cannot be nulled simultaneously in all cases.

**FIGURE 6:**
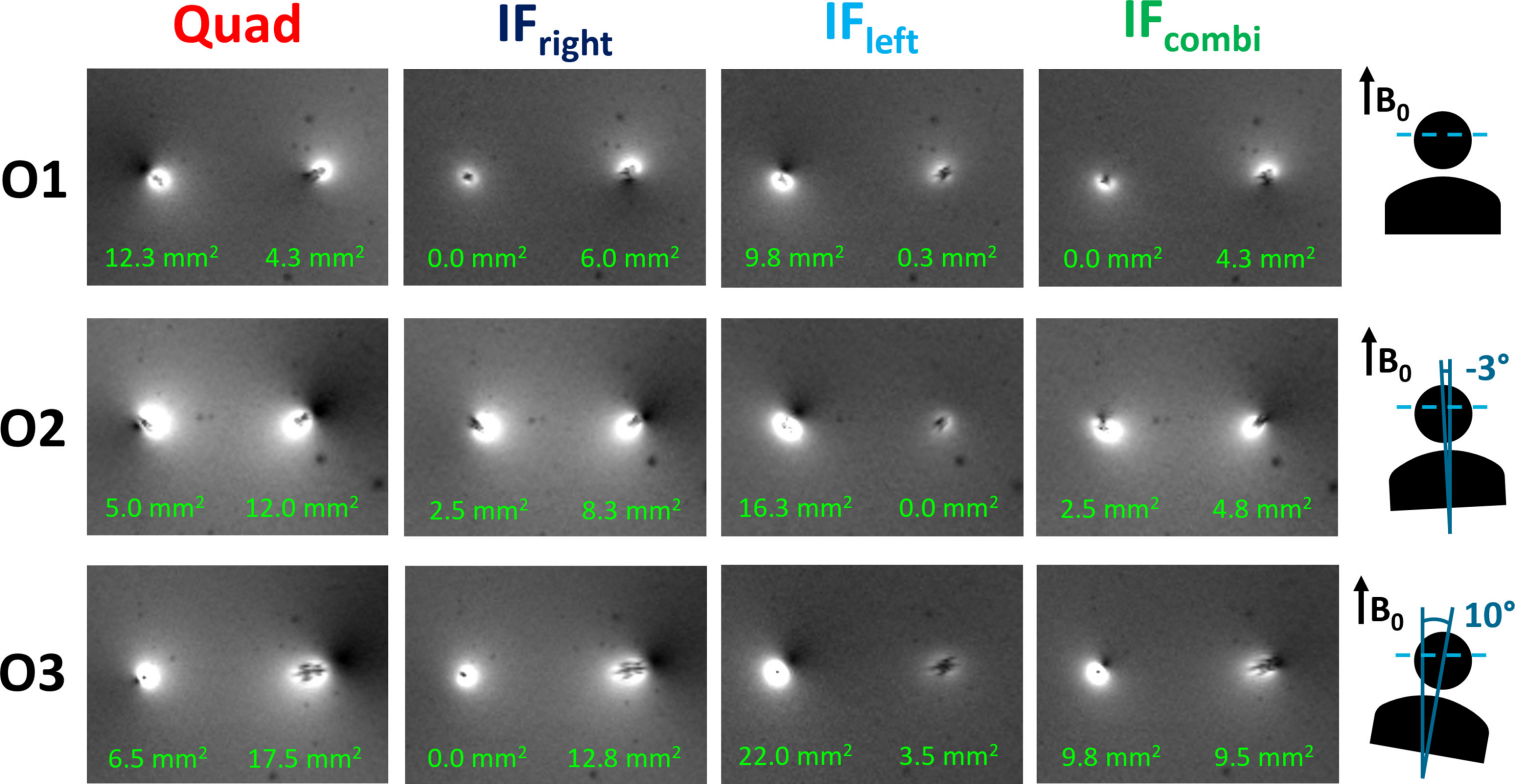
Gradient‐recalled echo scans in quadrature mode (Quad), the implant‐friendly modes of the right (IF_right_) and left lead (IF_left_), and the combined implant‐friendly mode (IF_combi_) across different orientations (O1–O3). The size of the Tx‐null artifact is indicated below each artifact. Schematic views of the top of the anthropomorphic phantom in different orientations are shown on the right, also indicating the direction of the *B*_0_ field and the angle between the *B*_0_ field and the central line between the leads. The patient right lead is indicated on the left and vice versa.

Figure 6 presents the results of the GRE scans and the corresponding Tx‐null artifact sizes acquired in quadrature, IF_right_, IF_left_, and IF_combi_ mode, across the different phantom orientations. In the IF_combi_ mode, for all orientations, excessive Tx‐null artifacts for either lead are prevented, due to a mitigation of the RF‐induced current. When comparing the IF_combi_ mode to the quadrature mode, the most severe Tx‐null artifacts in the quadrature mode are reduced. The most pronounced Tx‐null artifacts in the IF_left_ and IF_right_ modes, visible around the leads that are not optimized for, were also reduced or similar to those in the IF_combi_ mode. These observations confirm that the IF_combi_ mode strikes a proper balance in minimizing the RF‐induced current on both leads simultaneously, in all shown cases.

**FIGURE 7:**
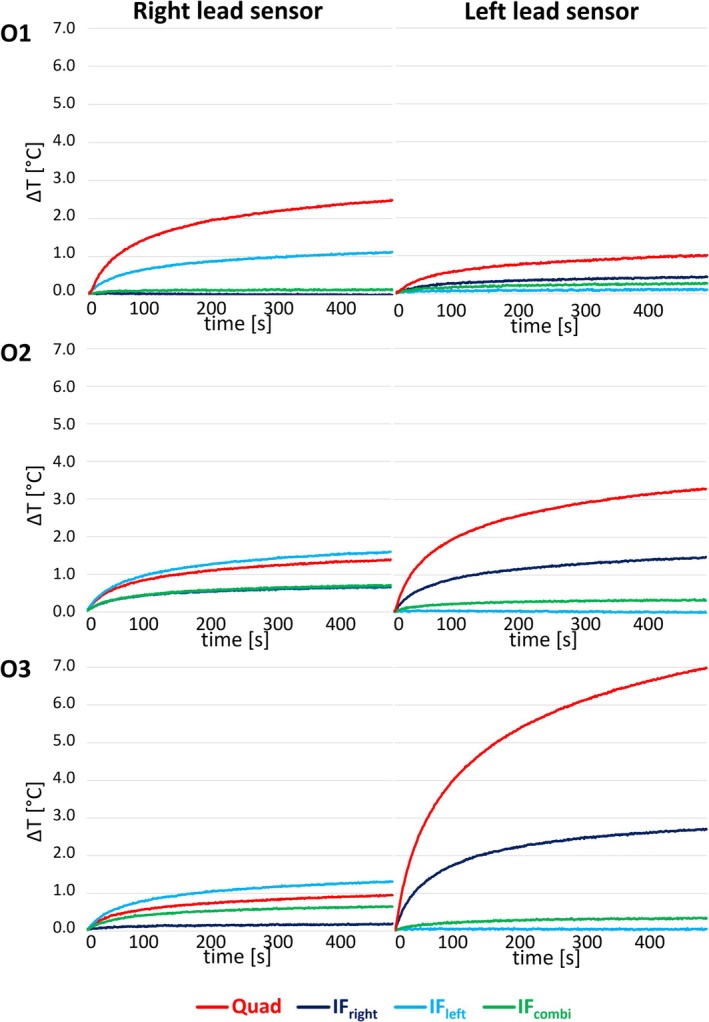
Heating curves measured during high‐SAR fast spin echo scans in quadrature mode (Quad), the implant‐friendly modes of the right (IF_right_) and left lead (IF_left_), and the combined implant‐friendly mode (IF_combi_) across different orientations (O1–O3).

Figure 7 and Table 2 depict the RF‐induced heating at both lead tips during the high‐SAR FSE sequence for the different phantom orientations, which corroborates the findings. Excessive heating in either lead is prevented in the IF_combi_ mode in all orientations. Additionally, the temperature increases observed in the IF_left_ and IF_right_ modes in the nonoptimized leads were either reduced or comparable to those observed in the IF_combi_ mode. RF heating results in quadrature mode demonstrated substantially increased heating compared to the IF_combi_ mode around both leads and for all three orientations, with increases reaching up to a factor of 11.4. This is associated with an increased Tx‐null artifact size in the corresponding images, as shown in Figure 6. These results highlight the efficacy and robustness of the workflow to minimize, rather than null, RF heating and reduce image artifacts in a bilateral DBS configuration using two‐channel RF shimming at 3T.

Throughout the experiments, the maximum temperature rise observed during the low‐SAR GRE calibration scans was 0.04°C ± 0.01°C in either lead. No measurable temperature rise was observed during any of the *B*_1_^+^ map calibration scans, nor during the multislice GRE scout scan.

**FIGURE 8:**
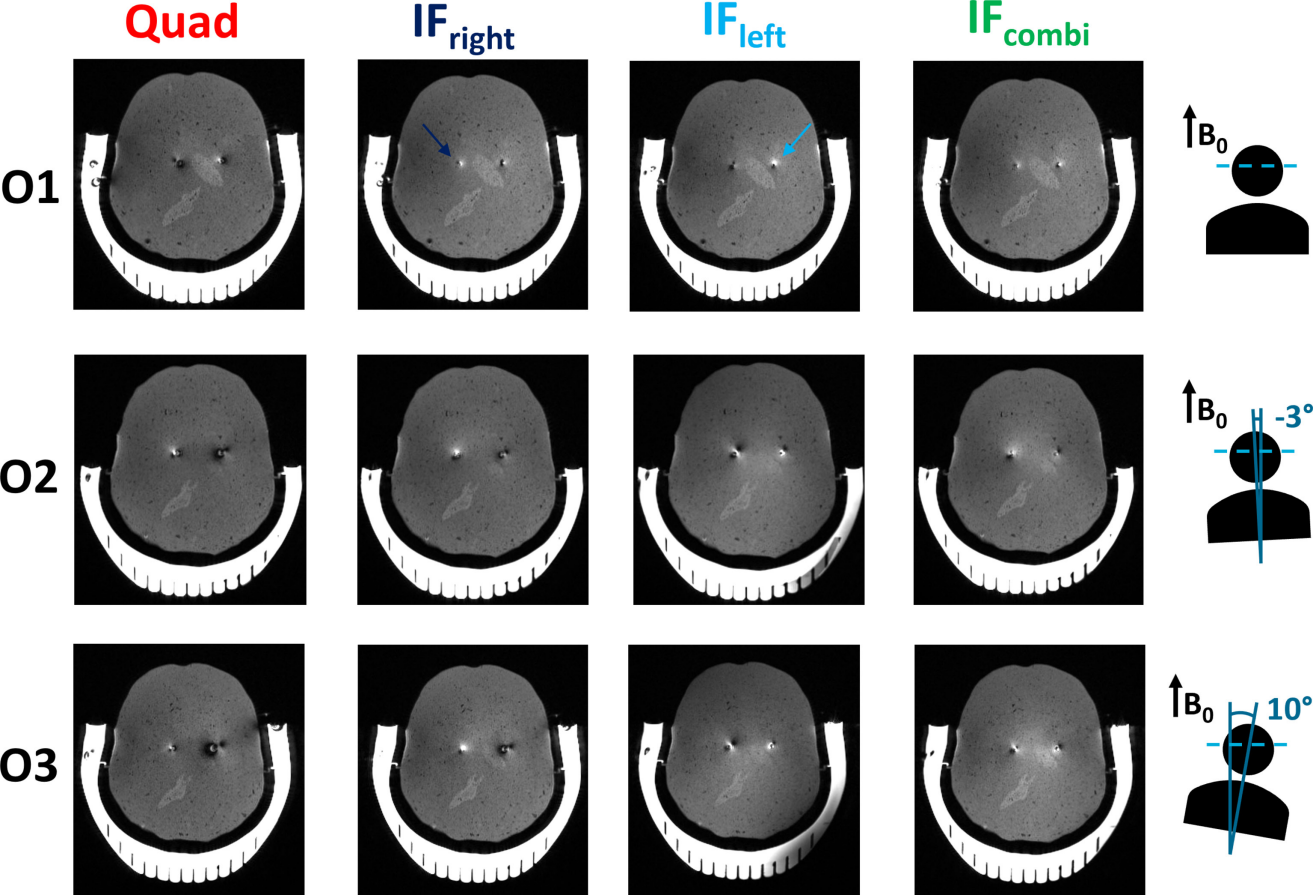
Fast spin echo scans in quadrature mode (Quad), the implant‐friendly modes of the right (IF_right_) and left lead (IF_left_), and the combined implant‐friendly mode (IF_combi_) across different orientations (O1–O3). The right and left leads for the respective implant‐friendly modes are indicated by an arrow in O1. Schematic views of the top of the anthropomorphic phantom in different orientations are shown on the right, also indicating the direction of the *B*_0_ field and the angle between the *B*_0_ field and the central line between the leads. The patient right lead is indicated on the left and vice versa.

As depicted in Figure 8, the FSE images acquired in IF_combi_ mode show benign image artifacts, confirming that the signal intensity loss around the lead is acceptable when compared with the images acquired in both IF_left_ and IF_right_ modes. Of the four RF shim modes, the most severe image artifacts were observed in quadrature mode, corresponding to the configuration with the highest RF heating. When visually comparing the overall image quality of the IF_combi_ mode to the quadrature mode, no degredation was observed.

## 4 Discussion

In this study, we evaluated the use of two‐channel RF shimming at a clinical 3T MRI scanner to minimize RF heating in a bilateral DBS lead configuration. Our approach exploits the geometrical relation between the location of the Tx‐null artifact with respect to the lead and RF heating, which are linked via the RF‐current induced on the lead. In our optimization method, we maximized the *B*_1_^+^ magnitude with respect to the largest of the RF‐induced currents on either DBS lead, thereby minimizing the peak RF heating around the lead tip and simultaneously improving image quality. Our experiments confirmed that this approach reduces RF heating when compared to both quadrature mode as well as to nulling the current on either lead, across all tested orientations. Moreover, overall image quality was preserved under the optimized RF shim conditions. A key distinction of our method lies in its robustness, achieved through the use of four low‐SAR calibration scans. Furthermore, this strategy is distinct from previous methods as it utilizes a clinically available two‐channel RF transmit architecture, avoiding the need for specialized systems with a higher number of transmit channels or dedicated RF transmit coils [39, 41, 52, 53].

Our findings indicate that it is possible to reduce the RF heating while maximizing the available *B*_1_^+^ field strength using a clinically available system. This could be crucial for ensuring MRI safety while scanning patients with a bilateral DBS lead configuration. The low‐SAR calibration scans showed minimal impact on the RF heating around the DBS electrodes, suggesting that our approach is likely to be safe for clinical use. Additionally, the proposed method may provide a strategy to enable configuration‐specific safety constraints, allowing for direct visualization of both the DBS lead and the target neuroanatomical structures within a single imaging modality. This may enhance the precision and reliability of postoperative DBS lead localization.

We note that the residual Tx‐null artifacts (Figure 6) showed some variability between the different orientations. Correspondingly, Table 2 highlights that the RF shim settings for the IF_left_, IF_right_, and IF_combi_ modes varied between the tested orientations. For example, the remaining artifact in IF_combi_ mode around the right lead is larger in orientation O3 with respect to orientation O1. This was accompanied by a higher temperature increase at the right lead in IF_combi_ mode in O3 (Figure 7 and Table 2). Although the difference in temperature increase cannot be directly linked to the variation in artifact size in all cases, since it is at least partially influenced by differences in average RF power and *B*_1_^+^_,rms_, both IF_combi_ modes effectively mitigated excessive heating and severe artifacts. These findings emphasize the robustness of the proposed method and highlight the importance of tailoring the optimal RF shim settings to specific lead configurations and orientations. Notably, the proposed method was able to detect the DBS lead even when its appearance was degraded, such as the smeared‐out presentation of the lead on the patient left side in orientation O3. This further demonstrates the method's resilience under various orientations and supports its applicability in clinical scenarios where lead visualization may be suboptimal.

In orientation O2, the RF shim that nulled the induced current on the right lead (i.e., IF_right_) resulted in a small residual Tx‐null artifact around the corresponding lead (2.5 mm^2^ in size) (Figure 6) as well as some noticeable residual heating during the high‐SAR FSE sequence (0.63°C ± 0.01°C) (Figure 7 and Table 2) compared with the RF shim optimized for the left lead (i.e., IF_left_). This might have been caused by acquiring the low‐SAR calibration scans perpendicular to the *B*_0_ field instead of acquiring them in a plane orthogonal to the lead [45]. However, it is most likely due to a residual phase difference between the incident *B*_1_^*+*^ fields provided by the system. This mismatch may be correlated to inhomogeneities present in the incident *B*_1_^+^ fields, as reported by CV in Figure 2A. However, the relation between the two is not fully consistent and is only known to become more relevant as the number of channels increases [52]. Nonetheless, this implies that the IF_right_ mode could have induced more heating (and a larger Tx‐null artifact) around the left lead, which was not optimized for, in the absence of this error. Even when comparing the IF_combi_ mode to this suboptimal IF_right_ mode, the IF_combi_ mode demonstrated substantially smaller artifacts and reduced heating around either lead.

Several potential limitations of the current approach may be considered. The proposed pixelwise division of a Gaussian filtered and a median filtered image might be less effective in a human subject with heterogeneous tissue contrast, compared with the phantom with homogeneous tissue contrast, in detecting the Tx‐null artifact. However, as can be seen in Figure S1, the low‐SAR GRE calibration scans showed little contrast inside a human brain of a healthy volunteer. The contrast ratio for the different RF shim settings of the white matter, through which the DBS leads will ascend, was 0.02–0.15 with respect to the cerebrospinal fluid and −0.22 to −0.32 with respect to the grey matter. In the images depicting the ratio between the Gaussian filtered and median filtered images in the human subject with heterogeneous tissue contrast, only the skull stands out. Nonetheless, in clinical practice, DBS leads are not placed near the skull, and for the outlier detection, the lead location must first be manually indicated. Therefore, it is anticipated that heterogeneous tissue contrast has minimal impact on the detection of the DBS leads and Tx‐null artifacts, making the semi‐automatic approach suitable for human subjects as well.

Another possible limitation of the proposed method is the possibility that a Tx‐null artifact, generated locally by the DBS electrodes, at a short distance from the lead, is obscured by susceptibility artifacts produced by the lead electrodes. The likeliness is reduced by characterizing the induced current at a location higher up along the lead, at 30 mm from the tip as proposed in Eryaman et al. [38], since these artifacts are minimal around the shaft of the electrode. This is similar to the susceptibility effects around the tip of a longitudinally oriented needle [54].

Our current findings warrant further exploration of the use of two‐channel RF shimming to address RF heating in bilateral DBS configurations. In this study, we focused on lead orientations and lengths commonly used in clinical practice for bilateral DBS implantation for Parkinson's disease. Although extracranial lead trajectories are known to affect RF heating [25], they were beyond the scope of the current work. The configuration‐specific nature of our approach suggests potential adaptability to other lead geometries, though this remains to be validated. Future studies could expand on this work by validating this approach in additional phantom setups, particularly mimicking markedly different DBS geometries and other target regions where a bilateral lead configuration is commonly used. Moreover, it would be beneficial to translate the residual level of RF‐induced current and associated heating into patient‐specific safety constraints, for example, patient‐specific SAR or *B*_1_^+^_,rms_ limits, with which a clinical MRI protocol could be established.

## 5 Conclusion

Two‐channel RF shimming on a clinical MRI scanner can be employed to minimize RF heating in a bilateral DBS lead configuration while preserving image quality, enabling safe imaging with minimal image artifacts. This approach could potentially serve to establish a patient‐specific workflow for safe imaging in patients with bilateral DBS leads at 3T.

## Author Contributions

C.V.S., W.R., and W.B. conceptualized and designed the study. E.A. constructed the phantom, and C.V.S., W.R. and W.B. developed the analysis tools. Data collection was performed by C.V.S. and W.B., and C.V.S. conducted the analysis. C.V.S. and W.B. drafted the paper with input from all authors.

## Supporting information


**Figure S1** The gradient‐recalled echo calibration scans for the anthropomorphic phantom with deep brain stimulation leads and a healthy volunteer without deep brain stimulation leads. Their respective linear incident *B*_1_^+^ field polarizations are indicated by the arrow. The ratio between the Gaussian filtered images and the median filtered images, necessary for the outlier detection method, are provided as well. The images indicate that little influence of the heterogeneous tissue contrast on the semiautomatic method for analyzing the GRE images can be expected. It is important to note that in clinical practice, leads will not be placed close to the skull.

## Data Availability

The data that support the findings of this study are available from the corresponding author upon reasonable request.
