# Supplementary material for: Reduction of RF Heating Near Bilateral Deep Brain Stimulation Leads Using Two‐Channel RF Shimming at 3T
Source: NMR Biomed. 2025 Aug 31;38(10):e70129. doi: 10.1002/nbm.70129 (PMC12399923; doi:10.1002/nbm.70129)

Supplementary materials

## Supplementary materials figure captions

Figure S1. The gradient-recalled echo calibration scans for the anthropomorphic phantom with deep brain stimulation leads and a healthy volunteer without deep brain stimulation leads. Their respective linear incident *B*_1_^+^ field polarizations are indicated by the arrow. The ratio between the Gaussian filtered images and the median filtered images, necessary for the outlier detection method, are provided as well. The images indicate that little influence the heterogeneous tissue contrast on the semi-automatic method for analyzing the GRE images can be expected. It is important to note that in clinical practice, leads will not be placed close to the skull.

## Supplementary Figure S1


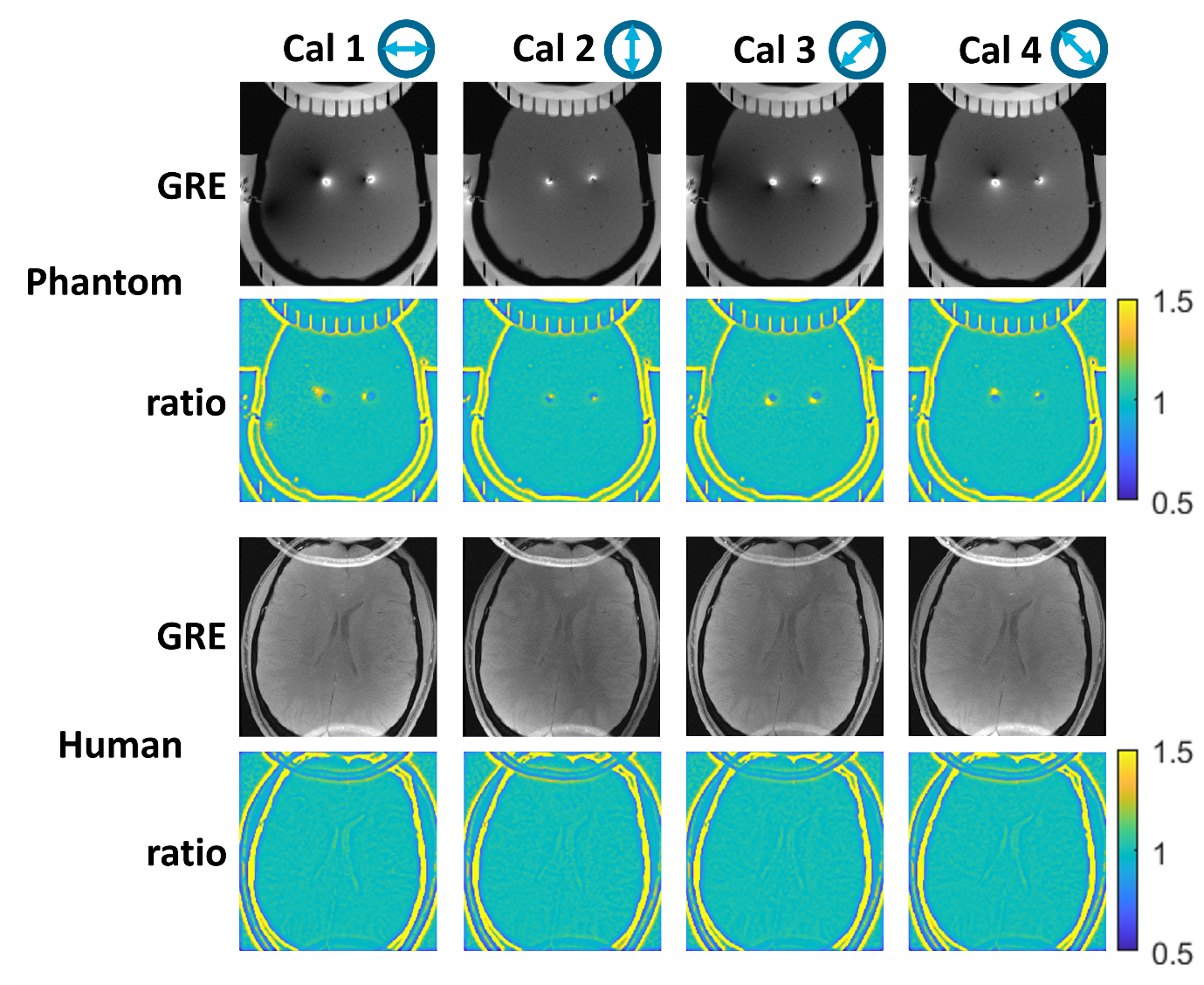

Supplement: Supplementary file 1 — Figure S1 The gradient‐recalled echo calibration scans for the anthropomorphic phantom with deep brain stimulation leads and a healthy volunteer without deep brain stimulation leads. Their respective linear incident B 1 + field polarizations are indicated by the arrow. The ratio between the Gaussian filtered images and the median filtered images, necessary for the outlier detection method, are provided as well. The images indicate that little influence of the heterogeneous tissue contrast on the semiautomatic method for analyzing the GRE images can be expected. It is important to note that in clinical practice, leads will not be placed close to the skull. [file NBM-38-e70129-s001.docx]
